# State-of-the-Art and Perspectives on Silicon Waveguide Crossings: A Review

**DOI:** 10.3390/mi11030326

**Published:** 2020-03-20

**Authors:** Sailong Wu, Xin Mu, Lirong Cheng, Simei Mao, H.Y. Fu

**Affiliations:** Tsinghua-Berkeley Shenzhen Institute, Tsinghua University, Shenzhen 518055, China; wusl17@mails.tsinghua.edu.cn (S.W.); mux17@mails.tsinghua.edu.cn (X.M.); clr18@mails.tsinghua.edu.cn (L.C.); maosm19@mails.tsinghua.edu.cn (S.M.)

**Keywords:** silicon photonics, integrated optics, waveguide crossing design, multimode interference (MMI), sub-wavelength grating (SWG), multiplexing technology

## Abstract

In the past few decades, silicon photonics has witnessed a ramp-up of investment in both research and industry. As a basic building block, silicon waveguide crossing is inevitable for dense silicon photonic integrated circuits and efficient crossing designs will greatly improve the performance of photonic devices with multiple crossings. In this paper, we focus on the state-of-the-art and perspectives on silicon waveguide crossings. It reviews several classical structures in silicon waveguide crossing design, such as shaped taper, multimode interference, subwavelength grating, holey subwavelength grating and vertical directional coupler by forward or inverse design method. In addition, we introduce some emerging research directions in crossing design including polarization-division-multiplexing and mode-division-multiplexing technologies.

## 1. Introduction

Towards the increasing demands of higher data rates, the issues of electric signal attenuation and power dissipation rise dramatically due to the intrinsic limitation of the parasitic effects in current metallic interconnection [1]. Moore’s law, the principle that has powered the information-technology revolution since the 1960s, is approaching its growth limit [2]. Fortunately, photons that have zero rest mass and zero charge, and can travel at the speed of light without interfering with electromagnetic field, is nearly 1000 times faster than electrons. The photonics integrated circuits (PICs) have greater advantages over the traditional integrated circuits (ICs) including higher data rate, larger bandwidth and lower power consumption. Hybrid technologies utilizing both PICs and ICs are advancing, enabling next generation science and technology for information society, pushing the boundaries of what is possible for telecommunications, computing, defense and consumer technology [3,4,5].

Silicon is a semiconductor that can both conduct electrons as a conductor or function as an insulator by controlling the charge and number of activated carriers in the doping processes, which makes silicon an ideal material to become the basis of memory chips, powering various devices from portable calculators to supercomputers. On the other hand, silicon is the second most abundant element on the earth. Besides, high-yield, large-scale silicon electronic devices can be manufactured with the current mature complementary metal-oxide semiconductor (CMOS) techniques. In terms of optical property, silicon is transparent for wavelengths from 1.1 to 8 μm, which covers near-infrared (NIR) and parts of mid-infrared (MIR) region [6]. The NIR covering the entire original-band (O-band) and conventional-band (C-band) with extremely low attenuation has been well studied for current fiber-optic communications. The MIR silicon photonics, which includes both atmospheric window (3–5 μm) and absorption bands (2.6–2.9 μm and >3.6 μm) of most chemical and biological molecules, as well as fingerprint region has recently been studied for optical interconnect and spectroscopic sensing, respectively [7,8]. As a result, silicon is regarded as an excellent candidate for the marriage of PICs and ICs on the same CMOS platform. The term “silicon photonics” refers to the applications of photonic systems which use silicon as an optical or sensing medium [9,10,11]. 

Silicon photonics, named by Soref, can date back to mid-1980s and began commercialization by Bookham Technology Ltd. in 1989 [12,13]. An explosive developments of silicon photonics have been witnessed in recent decades, revolutionizing a number of application areas, for example, data centers, high-performance computing and sensing [14,15]. The silicon-on-insulator (SOI) platform is promising due to its high refractive index contrast and the compatibility with commercial CMOS technology. In order to design dense and fully functional photonic components on a SOI platform, silicon waveguide crossing is critical and inevitable when the system is becoming more and more complexed, while, on the other hand, a compact device footprint is increasingly in demand. For electrical circuits, most of the printed circuit boards (PCBs) have four to eight layers and supercomputers typically contain boards with more than sixteen layers to improve the computation functionalities [16]. Controlling the PCB layers is a very flexible and mature technique for balancing the device performance and power consumption. However, efficient optical vias for multiple layers are very difficult to implement in the high-index contrast SOI platform. Therefore, this method cannot be employed in the silicon photonic circuits due to the limitations of optical mode coupling and fabrication cost [17]. For a typical direct silicon waveguide crossing design, the insertion loss is around 1.4 dB and the crosstalk is −9.2 dB [18], which means that the optical power nearly shrinks into a half after passing through only two cascaded silicon waveguide crossings. This kind of inefficient waveguide crossing design will greatly aggravate the performance of the advanced PICs devices with many cascaded waveguide crossings involved, such as optical routers [19,20]. In this review paper, we discuss and summarize different kinds of silicon waveguide crossing designs (e.g., shaped taper, multimode interference, subwavelength grating and vertical directional coupler structure). In addition, we introduce several recent hot research topics for the waveguide crossing design (e.g., polarization-multiplexing technology and mode-multiplexing technology). 

## 2. The Key Technologies of Silicon Waveguide Crossing

**Figure 1:**
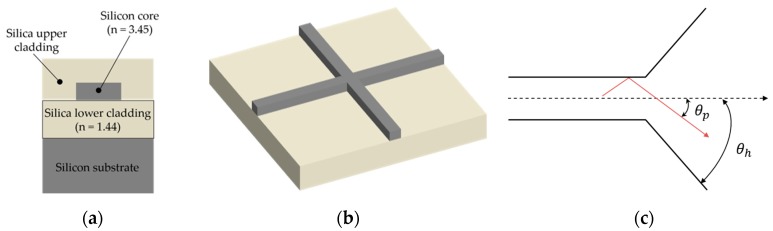
(**a**) Cross section of a typical silicon photonic wire waveguide. The grey structure represents the material silicon and the tawny one represents the material silica. The following schematic figure obeys the same rule. (**b**) Scheme of the direct silicon waveguide crossing on silicon-on-insulator (SOI) platform. (**c**) The ray model of the fundamental mode in the waveguide. $\theta_{p}$ is the light projection angle and $\theta_{h}$ is the horn angle of the optical waveguide.

Silicon waveguide usually consists of a 2 µm silica lower cladding, 220 nm silicon core and silica upper cladding on the 200 mm (8 inch) wafers, as shown in Figure 1a. It is fabricated by the deep ultraviolet (DUV) lithography and this technique can provide fast and reliable patterns. The refractive indexes of silicon and silica are 3.45 and 1.44, respectively, at 1550 nm, and the high refractive index contrast between the core and cladding results in strong optical confinement and ultra-small bending radius for fundamental modes [21]. The dimension of silicon core fulfills the optical single-mode condition and the experimental propagation losses of the fundamental mode are 2.4 ± 0.2 and 0.59 ± 0.32 dB/cm for transverse-electric (TE) and transverse-magnetic (TM) modes, respectively [22,23]. For the low refractive index contrast structure, such as optical fiber, direct waveguide crossings are just a minor perturbation of the straight waveguide [24]. However, side effects of the direct waveguide crossings cannot be ignored in the SOI platform for the beam is dramatically diffracted in the silicon intersection region, as shown in Figure 1b.

These phenomena can be approximately explained by the mode–conversion relationship between ray angle of the light and horn angle of the waveguide [25], as shown in Figure 1c. The silicon waveguide allows a total internal reflection of over 60° incidence angle due to the large refractive index contrast, while the horn angle jumps abruptly to 90° at intersection region for the direct waveguide crossing design. Huge mismatch between the projection angle and horn angle cannot maintain a smooth power transition. The large portions of light are excited into leaky higher-order modes and propagate into the vertical waveguides at the core region, resulting in the side effects of crosstalk and large insertion loss. From another point of view, the light is kept well confined in the silicon waveguide channel due to high refractive index difference before entering the “dangerous” core section. Once entering the intersection region, the horizontal silica cladding suddenly disappears and the optical power begins to scatter in all directions, resulting in severe effects of insertion loss, crosstalk and back-reflection. This section is organized to introduce several silicon waveguide crossing designs and explains their principles for improving the crossing performance.

### 2.1. Shaped Taper Method

Shaped taper waveguide crossing is to tailor the width of the silicon channel towards the center region, which decreases the diffraction effects by avoiding the shape mutation between the channel waveguide and crossing region. Shaped taper design includes different mathematic types, such as linear taper, parabolic taper, exponential taper and Gaussian taper [26]. With the shaped taper structure, the guided modes are expanded and the wide-angle spatial components are reduced, since the widths of waveguides are smoothly getting larger [27]. The diffraction side effects can be greatly controlled when the optical modes have fewer wide-angle spatial components. 

**Figure 2:**
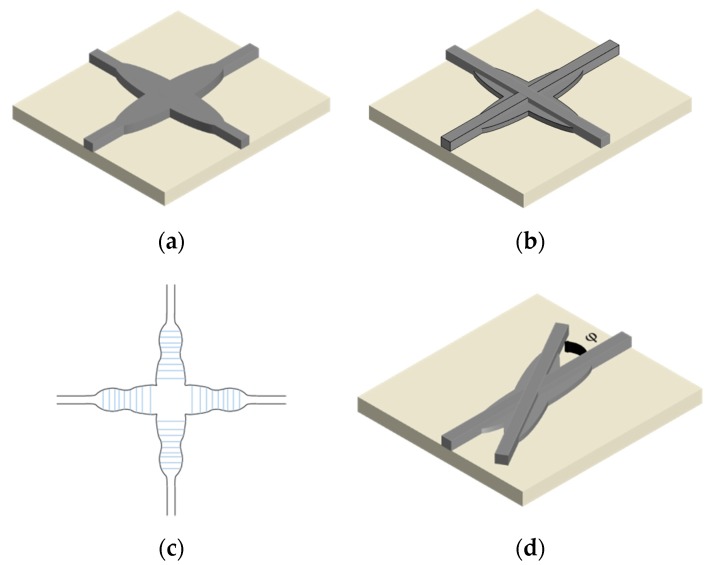
Schematics of (**a**) the shaped taper waveguide crossing and (**b**) the double-etched shaped taper silicon waveguide crossing; (**c**) the top view of the shaped waveguide crossing designed by the genetic algorithm (GA); (**d**) Schematic of the titled double-etched silicon waveguide crossing, φ represents the crossing angle.

The elliptical taper profile is used to get a narrow angular spectrum of the expanded mode, as shown in Figure 2a. The insertion loss and crosstalk of the device are <0.1 and <−30 dB with a footprint of 7.2 × 1.5 μm^2^, respectively [18]. For the nonadiabatic taper, the higher-order leaky optical modes will be excited and a sizable fraction of the power will be radiated away in the core region. The adiabatic taper can smoothly expand the guided modes, while the large taper footprint is not preferred for highly integrated PICs. As a result, a trade-off between its optical performance and footprint needs to be carefully considered. The double etching scheme consists of the high-contrast photonic wires in the upper level and the shadow-etched parabolic tapers in the lower level, as shown in Figure 2b. The confinement of the high refractive index contrast is maintained in the upper part and the lower parabolic expanded tapers play a role to adjust the optical phase fronts. The double DUV etching scheme waveguide crossing can greatly shrink the footprint to 6 × 6 μm^2^ and the insertion loss and crosstalk are 0.16 and −40 dB, respectively [28]. However, the additional DUV stepper lithography increases the fabrication complexity and cost of the dual-etching device are much higher than that of the conventional single DUV etching process, which are the main limitations of this kind of waveguide crossing design. Different from the assumed mathematical taper profile with only a few parameters to be determined, a numerical optimization method has more degrees of freedom by separating the entire taper into many spaced segments with different widths, as shown in Figure 2c. The shaped taper can be formed by connecting all the segments with the spline function and the optimization process is implemented to minimize the insertion loss and crosstalk by tuning the widths of all the segments with the help of advanced algorithms. The insertion loss and crosstalk are <0.2 and <−40 dB, respectively. The footprint is 6 × 6 μm^2^ for the silicon waveguide crossing designed by genetic algorithm (GA) [29]. For particle swarm optimization algorithm (PSO) assisted silicon waveguide crossing design, the insertion loss and crosstalk are −0.0278 ± 0.0092 and <−37 dB, respectively. The device has a footprint of 9 × 9 μm^2^ [30]. However, DUV steppers are not designed for high resolution purposes and the performance begins to degrade for small designs. The small numerical optimized shaped taper can be fabricated with single etch process, while the fabrication tolerance is small since the performance is sensitive to the device geometry. The design performance varies greatly in terms of the fabrication errors in DUV technology and this is the main drawback for this crossing design. For the conventional silicon waveguide crossing, the crossing angle is 90° and the device is consisted of two perpendicular arms. Researchers find that the crosstalk can be improved by more than 10 dB without degrading transmission losses where the crossing angle is set to be 60° for the direct waveguide crossing. For the double-etched waveguide crossing, the crosstalk can be reduced by 3.7 dB without degrading transmission losses by titling waveguide crossing angle to be 60° [31], as shown in Figure 2d. The offset crossing with a small angle of 20° is also proposed and proves to be beneficial for further reducing the crosstalk [32].

### 2.2. Multimode Interference Method

The structure of the multimode interference (MMI) device is such that single-mode waveguide connects to both sides of a multimode waveguide, and there exists the self-imaging phenomena that an input signal pattern is replicated, at periodic intervals, once or multiple times along the direction of propagation along the waveguide [33]. If the light is launched at the center position of the waveguide, only the even symmetric modes will be excited and a symmetric field profile is obtained by the linear combinations of the even modes [34]. For the symmetric field interference, the light from the single-mode waveguide firstly diverges in the first half and focus at the middle-central position where the replicated optical spot size from the single-mode waveguide is much smaller than the MMI waveguide cross-section. Then the light diverges in the next half of the MMI section and recouples to the single-mode waveguide. The self-imaging property in the MMI structure proves to be robust where the lateral confinement is relieved in the silica waveguide crossing design, which is quite suitable for the waveguide crossing design [35]. 

**Figure 3:**
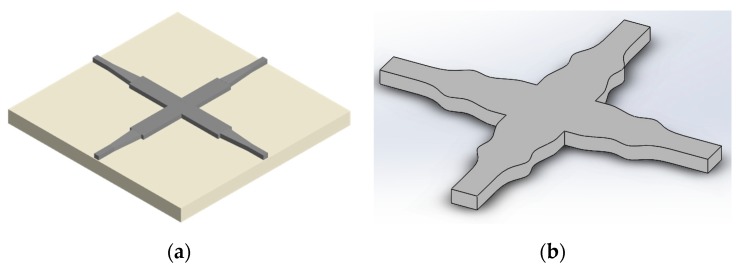
Schematics of (**a**) the traditional multimode interference (MMI) silicon waveguide crossing with taper transition and (**b**) the three cascaded multimode tapers silicon waveguide crossing.

A typical MMI silicon waveguide crossing consists of four single-mode arms and two multimode waveguides supporting both TE_0_ and TE_2_ modes, as shown in Figure 3a. The tapers connecting single-mode and multimode waveguides are usually employed to reduce the back-reflection at the intersection region. The insertion loss and crosstalk of the tapered MMI silicon waveguide crossing are ~0.4 and <−30 dB, respectively. It has a footprint of 13 × 13 μm^2^ [36]. In addition, the length of the MMI core can be reduced to less than 6 μm with a sophisticated taper by matching the Gaussian beam pattern with 0.21 dB insertion loss and −44.4 dB crosstalk [37]. Another method to fulfill the phase difference requirements between the two lowest even modes is to employ three cascaded multimode tapers, and the insertion loss is 0.13 dB and the crosstalk is −43.5 dB at the footprint of 4.16 × 4.16 μm^2^ [38], as shown in Figure 3b. The symmetric MMI crossing supporting TE_0_, TE_2_ and TE_4_ modes with wider MMI waveguides and the synthesized Gaussian-like focusing pattern mode has an extremely low insertion loss of 0.007 dB ± 0.004 dB and crosstalk of <−40 dB at a footprint of 30 × 30 μm^2^ [39]. The low-loss Bloch waves can be viewed as the combination of the multimode self-focusing property with the matching of the field pattern and dielectric structure periodicities, which have low insertion loss of 0.045 dB and crosstalk of −34 dB [40]. However, the Bloch wave waveguide crossings have low fabrication tolerance and the mismatched periods can cause 0.65 dB loss, which is 15 times larger than that of the matched periods. The self-imaging property can also be employed in the silicon-based slot–waveguide crossing with insertion loss of 0.086 dB and crosstalk of −27.51 dB [41]. Similar to the shaped taper waveguide crossing, the crosstalk of MMI waveguide crossing with 110° crossing angle can be improved by more than 14 dB compared with the conventional waveguide crossing with 90° crossing angle [42]. On the other hand, the multiple ports silicon star-like crossings can greatly improve the system capacity compared with the traditional 2 × 2 crossing design [43,44,45]. The MMI-based star-like 3 × 3, 4 × 4, 5 × 5 and 6 × 6 silicon crossings are proposed and have very low insertion loss and crosstalk for all propagation channels [46]. MMI waveguide crossing is robust against the fabrication errors because the large waveguide width in MMI design is very suitable for the mature DUV technique. MMI waveguide crossing is the most popular waveguide crossing design in PICs for industry. However, the device footprint is relatively larger compared with other crossing designs, which limits its applications for ultra-compact PICs.

### 2.3. Sub-Wavelength Grating Method 

Sub-wavelength grating (SWG) waveguide is a periodic arrangement of two different materials having a period that is shorter than the wavelength of light. The electromagnetic wave propagation in SWGs structures with dielectric and metal layers have been theoretically studied since 1943 [47,48]. The light propagation in the SWG waveguide can be divided into three operation regimes, including reflection regime, diffraction regime and subwavelength regime. The periodic waveguides are often employed as distributed Bragg reflector (DBR) lasers in reflection regime while as grating couplers in diffraction regime [49,50,51]. In the subwavelength regime, the diffraction and reflection effects are eliminated and the SWG waveguide can be treated as an equivalent homogeneous medium with an approximately effective refractive index given by Rytov, which is widely used in SWG waveguide crossing design. 

**Figure 4:**
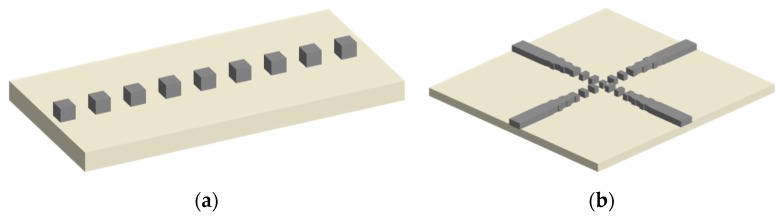
(**a**) Schematic of the sub-wavelength grating (SWG) silicon waveguide and (**b**) top view of SWGs silicon waveguide crossing with tapers.

For SOI material platform, the SWG waveguide consists of a periodic arrangement of silicon and silica (air) layers and the effective refractive index can be tuned by chirping the duty cycle, pitch and tapering the width of the grating segments, as shown in Figure 4a. In the SWG waveguide, Bloch–Floquet optical mode is excited and its unique delocalizing property results in the efficient waveguide crossing [52]. The modal optical confinement is partly maintained for the SWG unique structure at the intersection region and the diffracting loss is reduced compared with the direct waveguide crossing [53]. On the other hand, the effective refractive index of SWG waveguide is smaller than that of the conventional silicon channel, and the scattering portion is not as strong as that in the relatively low refractive index waveguide crossing. SWG is intrinsically birefringent and the refractive indexes of the parallel and perpendicular directions are different, which can be beneficial for designing a polarization-insensitive waveguide crossing for both TE and TM modes. For the SWG waveguide crossing design, it has impressive insertion losses of 0.02 and 0.04 dB for TE and TM polarizations, respectively, and crosstalk below −40 dB with single etch fabrication step [54]. To reduce the mode mismatch introduced loss and prevent back reflection at the interface, SWG taper is often used to couple light from a conventional strip waveguide to the SWG waveguide by gradually reducing the waveguide width for fulfilling the effective index matching condition [55], as illustrated in Figure 4b. However, the structure requires ~10 × 10 μm^2^ large adiabatic taper and an induced 0.3 dB loss per taper, which are the main drawbacks of this design. In another point of view, the SWG can be served as a refractive index engineering method and is flexible to be adopted in the mature crossing method, such as MMI crossing. E-beam lithography (EBL) is often employed due to the very small structures in SWG. EBL works by directing a beam of electros at the wafer exposing an EBL resist point by point, which is much slower than the DUV technique. The silicon waveguide crossings using the lateral index-engineered cascaded multimode-interference couplers are proved to have <0.01 dB insertion loss and <−40 dB crosstalk [56]. Owning to the compact size of MMI crossing, the index-engineered MMI coupler waveguide crossing can further reduce the footprint to around ~3 × 3 μm^2^.

### 2.4. Holey Subwavelength Grating Method 

**Figure 5:**
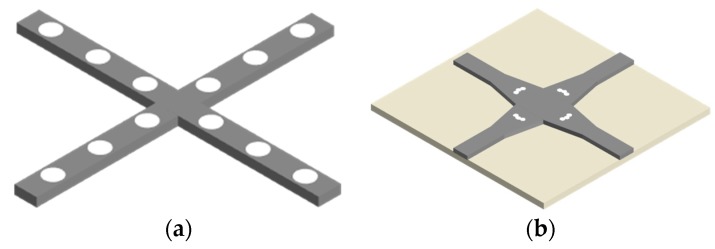
(**a**) Schematic of the inverse-designed silicon waveguide crossing and (**b**) top view of the ultra-compact silicon waveguide crossing with holey SWG grating method.

In recent years, numerical optimization methods are becoming more and more powerful and the single-layer inverse-designed structure is popular because the device can be easily divided into m × n pixels. Each pixel can have the assumed shapes, like square or circle, and the topology optimization process is to decide the presence or absence of these silicon pixels [57]. Figure 5a depicts the conventional inverse designed silicon waveguide crossing. To prevent the optical power diffracting into the transverse ports, the center of the intersection region and four arms are occupied by arrayed circular holes. The optical mode in the input port belongs to the resonant mode and the diffraction loss will be suppressed according to the phenomenon of resonant tunneling through a cavity with the insertion loss of ~0.2 dB [58,59]. However, the optical wavelength range is so restricted and it is impractical for the real applications. Different from the traditional etched holes, the four etched lens-like structures are placed before the intersection and form a waveguide guiding region in the crossing section, resulting in high power transmission and low crosstalk [60]. The device has a low insertion loss of <0.175 dB and low crosstalk <−37 dB with an extremely small footprint of ~1 × 1 μm^2^, which is the most compact silicon waveguide crossing to the best of our knowledge, as shown in Figure 5b.

The inverse-designed waveguide crossing is a refractive index engineering method and can modify the refractive effective index distribution of the device as wanted. In the design [61], the silicon waveguide crossing has a square-assembling pattern with 51 × 51 pixels and the dimension of each pixel is 100 × 100 nm^2^. The expensive EBL technique is required due to the ultra-small pixel size. The advanced algorithm determines the states of each pixel and engineer the refractive index distribution. The waveguide crossing has an insertion loss of 0.1–0.3 dB and crosstalk of <−35 dB at a footprint of 5 × 5 μm^2^. From the large scales of optical channels to intercross connects, like 6 × 6 crossing, the utilization of the traditional 2 × 2 waveguide crossing will be very inefficient and take up huge footprint. Some multiple-input multiple-output (MIMO) crossing designs have been proposed to increase the density of the ports in a given area. The 4 × 4 PhC star-like inverse-designed waveguide crossing is proposed with a nonlinear direct-binary-search (DBS) optimization algorithm, and the insertion loss is 0.75 ± 0.2 dB and the crosstalk is <−20 dB [62]. 

### 2.5. Vertical Directional Coupler Method

Different from the previous methods of realizing the efficient silicon waveguide crossing in a single layer, the vertical directional coupler method is used to couple light from the base silicon channel to the upper or lower optical waveguide and transfer the power back into the original silicon waveguide without coming through the “dangerous” crossing region. The vertical directional coupler method, also named the waveguide bridge method, has the lowest crosstalk in theory and the key consideration for this design is to realize the efficient power coupling in the limited device footprint together with an acceptable fabrication cost. 

**Figure 6:**
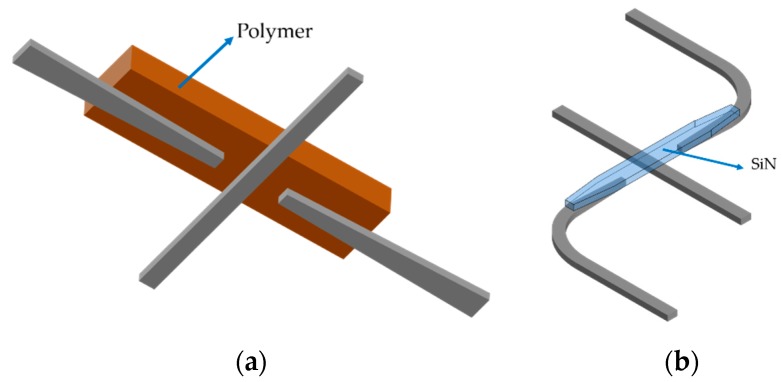
Schematics of the silicon waveguide crossing with vertical directional coupler consisting of (**a**) silicon and polymer layers and (**b**) silicon and SiN layers.

For the silicon and silica-based waveguides, the large difference in propagation constants is difficult for transferring the optical power between these two layers [63], which results in the inefficient direction coupler and causes a large device footprint. Vertical optical waveguide coupler consisting of SOI and amorphous silicon is proposed to solve this issue, and it has the insertion loss of 0.2 dB [64] in reasonable transition length while the fabrication cost is unacceptable. For the integration of silicon and polymer layers, the optical power is coupled up and down when passing through the silicon waveguide crossings and the performances are much better with 0.08 dB insertion loss and −70 dB crosstalk [65], as shown in Figure 6a. However, the dimension of polymer waveguide is much larger than the silicon waveguide due to the weak optical confinement and the bridge waveguide crossing takes up more space compared with the crossings in a single layer. The main drawback is that the laterally stacked three silicon optical waveguides are very complicated to fabricate and these fabrication techniques are not wildly used in the mature CMOS process. Silicon nitride (SiN) is probably the most promising material for the integration with the SOI platform due to its excellent CMOS fabrication compatibility and low propagation loss in the optical communication band. The SiN over Si bridge waveguide crossing has an extremely low insertion loss of −49 dB and crosstalk of −65 dB [66], as shown in Figure 6b. For multilayer SiN-on-Si integrated photonic platforms, bilevel and trilevel grating couplers are used in these three layers platforms and they have been demonstrated to have low-loss interlayer insertion loss and ultralow-loss crosstalk. 

The low-loss, low-crosstalk, compact and low-cost integrated silicon waveguide crossings are designed to satisfy the increasing demands of PICs in the coming era of the “BIG DATA” and the “Internet of Things” [67,68]. Table 1 summarizes the typical results of different silicon waveguide crossing design methods and includes the key information about the insertion loss, crosstalk, device footprint, silicon platform thickness and fabrication cost. These sophisticated silicon waveguide crossings are proposed to fulfill all these figure of merits (FOMs) and each structure has its unique advantages and disadvantages in these FOMs. For example, the vertical directional coupler waveguide crossing has probably the best crosstalk and insertion loss performance but is not popular for its expensive fabrication cost. In addition, the MMI waveguide crossing is widely acknowledged for its fabrication tolerance and moderate insertion loss and crosstalk performances, while it is limited by its relatively large device footprint. Different silicon waveguide crossing designs are employed in different PICs. 

## 3. The Future Trends of Silicon Waveguide Crossing

As there is an increasing demand for ultra-high capacity optical interconnects, different silicon-based multiplexing technologies have been investigated to achieve a high-data rate [69,70]. Polarization-division-multiplexing (PDM) and mode-division-multiplexing (MDM) technologies are becoming hot research topics, because they make it possible to increase the optical link capacity by introducing dual polarization states as well as several mode states in the optical channel [71]. Polarization dependent loss (PDL) and the mode dependent loss (MDL) become additional FOMs for the silicon waveguide crossing design. Polarization and mode insensitive silicon waveguide crossings are the future trends and have great potential to be employed in the dense photonic integrated systems. Table 2 summarizes the FOMs of silicon waveguide crossing designs with multiplexing technologies and detailed characteristics will be discussed in the following paragraphs.

### 3.1. Mode-Division-Multiplexing Technology 

Most traditional PICs are designed only for the fundamental optical mode for its characteristic of low loss and crosstalk. Higher order modes are attracting much more attention and are introduced to carry signals together with the fundamental modes to increase the link capacity for the PICs. The mode-division-multiplexing technology (MDM) is becoming more and more popular [78,79,80,81]. The sophisticated waveguide crossings used for the single-mode cannot be directly applied for higher order modes due to the different refractive indexes and optical power distributions, while the design principles and process may be similar to those in the single-mode waveguide crossing.

**Figure 7:**
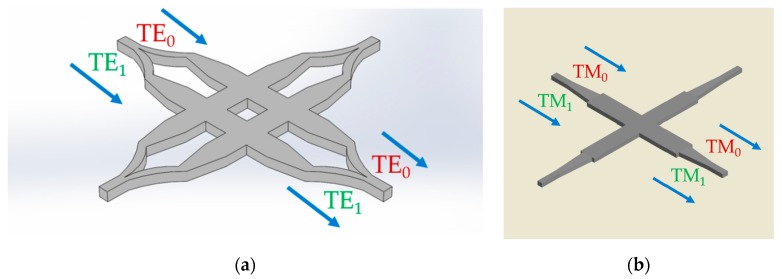
(**a**) Schematic of the mode-multiplexed silicon waveguide crossing with Y-junction mode splitter for fundamental transverse-electric (TE_0_) and first-order transverse-electric (TE_1_) mode; (**b**) top view of the mode-multiplexed silicon waveguide crossing with multimode interference structure for fundamental transverse-magnetic (TM_0_) and first-order transverse-magnetic (TM_1_) mode.

The most straightforward method is to separate the combined modes into different paths by using the mode splitter device, so the design of the single-mode waveguide crossing can be utilized in each optical path. Figure 7a shows the mode-multiplex silicon waveguide crossing with a Y-junction-based mode splitter. The TE_0_ and TE_1_ modes are separated and the traditional MMI silicon waveguide crossing is selected to form the efficient 2 × 2 crossing matrix [72]. The MMI length needs to be optimized to get a good balance between TE_0_ and TE_1_ modes, since there exists a gap between the beat lengths for those two modes. The device has the insertion loss of 1.82 (0.46) dB for TE_0_ (TE_1_) mode at 1550 nm and crosstalk of −18 dB with the device length of 23 μm. Another kind of efficient mode splitter is the PhC-assisted subwavelength asymmetric Y-junction structure and it can separate the TE_0_, TE_1_ and TE_2_ modes simultaneously [82,83]. Similarly, a 3 × 3 crossing matrix is achieved by the MMI waveguide crossing whose insertion loss is less than 0.9 dB and crosstalk is lower than –24 dB for all the three modes at the length of 34 μm [84]. The other category of MDM silicon waveguide crossing is not to separate the combined modes but to manipulate the optical modes in the same crossing design. The launched TM_0_ (TM_1_) mode is converted to the combination of TM_0_ (TM_1_) and TM_2_ (TM_3_) mode after the tapered waveguide and self-imaging property is applied to form the efficient waveguide crossing, as shown in Figure 7b. The drawback of this MMI is that its length usually expands to the least common multiple (LCM) between these two beat lengths, so it is hard to get good transmission performances for both TM_0_ and TM_1_ modes. This device has an insertion of around 1.5 dB and crosstalk of −18 dB for TM_0_ and TM_1_ modes at a length of larger than 55 μm. The same MDM crossing design can be employed for TE_0_ and TE_1_ modes. The combination of strip and rib waveguides can compensate the gap of the beat length for the two modes, and reduce the device footprint with the cost of double etch fabrication. The insertion loss is <0.87 (0.54) dB for TE_0_ (TE_1_) mode and the crosstalk is less than −50 dB for both modes at the device length of 33.7 μm. Different from the combination of strip and rib waveguides, the subwavelength MMI crossing can realize the identical beat length for TE_0_ and TE_1_ mode by engineering the refractive index with the inverse design method to get the complex refractive index distribution [85]. The device has an insertion of less than 0.6 dB and crosstalk of −42 dB for both TE_0_ and TE_1_ modes at a length of 4.8 μm, which is a very compact device footprint for the MMI-based crossing. This design has large scalability and can be redesigned to support more modes in the future. Like the polarization-multiplexing technology, the designing process of mode-division-multiplexing technology can also be viewed as a further step compared to the traditional silicon waveguide crossing design, since more than one optical mode needs to be considered. 

### 3.2. Polarization-Division-Multiplexing Technology 

TE mode is general used in silicon channel application due to its ultra-small bending radius as small as 2.5 μm, while a few tens of micrometers are needed for TM-like mode [86]. Polarization splitters and rotators (PSRs) are used in the polarization diversity systems [87,88,89]. For the previously proposed waveguide crossing designs, they are more focused on TE-like mode while TM-like mode has not attracted much attention. It is intuitive that PDM technology with dual polarizations is capable of doubling the link capacity in silicon waveguide, since optical polarization states can be intrinsically divided into two perpendicular polarization states, TE and TM polarizations [90]. However, the high structural birefringence of SOI platform introduces a large effective refractive index gap and an imbalanced performance of TE and TM polarizations in the previous proposed silicon waveguide crossing designs. 

**Figure 8:**
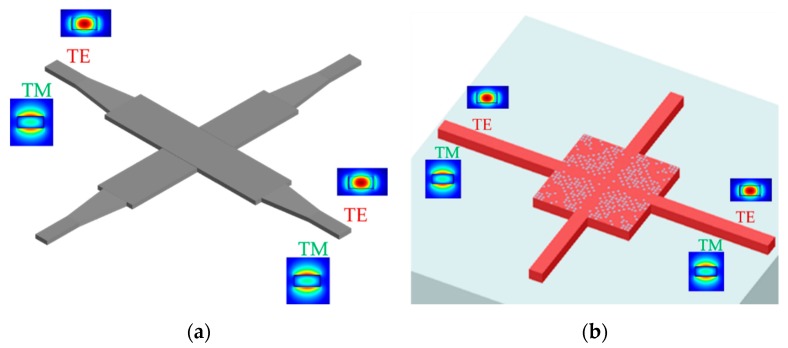
(**a**) Schematic of the polarization-multiplexed MMI silicon waveguide crossing; (**b**) top view of the polarization-multiplexed inverse-designed silicon waveguide crossing.

For the MMI silicon waveguide crossing design, the length of the MMI section is mainly determined by the optical beat length which depends on the effective refractive index of the optical mode. Unfortunately, the beat lengths for TE and TM polarizations are not the same and a trade-off needs to be made by expanding the device into the position near the LCM to get a good transmission performance for dual polarizations, as shown in Figure 8a. The polarization-insensitive silicon MMI crossing is proposed and has the insertion loss of 0.73 dB (0.65 dB) and crosstalk of −30 dB (−45 dB) for TE (TM) polarization with the device length of 23 × 23 μm^2^ at 220 nm SOI platform, which is a relatively large device footprint in the modern PICs. The polarization-insensitive silicon shaped taper waveguide crossing is demonstrated by using the numerical inverse design method and the device can achieve insertion loss of 0.08 (0.07) dB and the crosstalk of −32 (−35) dB for TE (TM) mode with the device length of 6 μm at 250 nm SOI platform. Previously, we have explained the theoretical principle of tuning the effective refractive index with the subwavelength grating method. However, the taper will introduce the additional insertion loss. We proposed a SWG-assisted MMI crossing and reduced the MMI-based device footprint by narrowing the refractive index gap between TE and TM polarizations with the SWGs structure [91]. The device has the insertion loss of 0.69 (0.61) dB and crosstalk of −45 (−35) dB for TE (TM) polarization at the moderate footprint of 12.5 × 12.5 μm^2^ on 220 nm SOI platform. For the holey SWG silicon waveguide crossing, the PDL is also taken into consideration as another designing targets, as shown in Figure 8b. The device can achieve the insertion loss of −0.67 (−0.69 dB) and less than –20 dB for TE (TM) mode at the extremely small footprint of 3.6 × 3.6 μm^2^ at 340 nm SOI platform. The designing principle and process keep roughly the same whether the waveguide crossing is polarization sensitive or not. The polarization-insensitive waveguide crossing adds another design constraint since the TM polarization mode is also needed to be considered.

### 3.3. Summary 

Silicon photonic waveguide crossing is a basic passive building block in PICs and the target is manipulating light to effectively go through the “dangerous” intersection region of structures, with features near or below the scale of the electromagnetic wavelength. The workflow is to design new crossing structures with some prior known physical effects in waveguide crossing, such as the optical coupling theory in taper, the self-imaging principle in multimode waveguide and so on. To meet the increasing requirements for the optical interconnect, the effective waveguide crossing is not only limited to low insertion loss and crosstalk but also needs to be integrated with different merits, such as the polarization insensitivity, mode multiplexing technology and ultra-compact footprint. On the other hand, inverse-design methods in nanophotonics are widely adopted by researchers and impressive progresses are achieved in different photonic devices [92,93]. 

## 4. Conclusions

In this paper, we reviewed the state-of-the-art and provided our perspectives on the silicon waveguide crossing. It gives the theoretical principle and device performance about several important waveguide crossing design methods such as shaped taper, multimode interference, subwavelength grating, holey subwavelength grating and vertical directional coupler. On the other hand, we also include some future trends of silicon waveguide crossing to meet the increasing requirements in data rates for PICs, like polarization-division-multiplexing and mode-division-multiplexing technologies. We believe that a boost in the number of silicon photonic products is coming to the market and an increase in the number of complex silicon photonic systems is being developed in both academia and industry. The silicon waveguide crossing is one of the basic building blocks in PICs and its development will pave the path of complex and functional PICs in the near future. 

## Figures and Tables

**Table 1:** Comparisons of different silicon waveguide crossing designs.

Type	Institute	Insertion loss (dB)	Crosstalk (dB)	Footprint (μm^2^)	Thick (nm)	Fabrication Cost	Ref.
Shaped taper	YNU^1^	<0.1	<−30	7.2×1.5	320	Low	[18]
Shaped taper	Ghent Uni.	0.16	−40	6×6	220	Low	[28]
Shaped taper	UPV^2^	<0.2	<−40	6×6	250	Medium	[29]
Shaped taper	Univ. of Delaware	~0.028	<−37	9×9	220	Medium	[30]
MMI	HKUST^3^	~0.4	−30	13×13	340	Low	[36]
MMI	NCTU^4^	0.13	−43.5	4.1×4.16	220	Low	[38]
MMI	Huawei	~0.007	< −40	30×30	220	Low	[39]
MMI	Southeast Uni.	0.086	−35.58	~16×16	250	Low	[41]
SWG	NRC^5^	0.023	<−40	~10×10	260	Medium	[54]
SWG	UT Austin^6^	~0.02	<−40	~3×3	250	Medium	[56]
Holey SWG	SYSU Uni.	0.1~0.3	<−35	~5×5	220	High	[61]
Holey SWG	HUST^7^	0.75	<−20	--	220	High	[62]
Vertical DC	ISP SB RAS^8^	0.08	−70	--	--	High	[65]
Vertical DC	SNL^9^	0.16	−49	--	--	High	[66]

1. Yokohama National University. 2. Valencia Nanophotonics Technology Center. 3. Hong Kong University of Science and Technology. 4. National Chiao Tung University. 5. National Research Council, Ottawa, Canada. 6. University of Texas at Austin. 7. Huazhong University of Science and Technology. 8. Rzhanov Institute of Semiconductor Physics of the Siberian Branch of the RAS, Russian. 9. Sandia National Laboratories, Albuquerque, USA.

**Table 2:** Comparisons of silicon crossing with mode-division-multiplexing (MDM) and polarization-division-multiplexing (PDM) technologies.

Type	Institute	Insertion Loss (dB)	Crosstalk (dB)	Footprint (μm^2^)	Thick (nm)	Ref.
MDM	HUST^1^	TE_0_: ~1.82 TE_1_: ~0.46	<−18	21×21	220	[72]
MDM	HUST	TE_0_: 0.87 TE_1_: 0.54	<−50	33.7×33.7	220	[73]
MDM	Zhejiang Uni.	TM_0_: 0.56 TM_1_: 0.84	<−20	~32×32	340	[74]
PDM	Zhejiang Uni.	TE_0_: 1.2 TM_0_: 1.5	<−25	23×23	220	[75]
PDM	CUHK^2^	TE_0_: 0.2 TM_0_: 0.5	<−28	6×6	250	[76]
PDM	TBSI^3^	TE_0_: 0.67 TM_0_: 0.69	<−20	3.6×3.6	340	[77]

1. Huazhong University of Science and Technology. 2. Chinese University of Hong Kong. 3. Tsinghua-Berkeley Shenzhen Institute, Tsinghua University.
